# In vivo and in silico screening for antimicrobial compounds from cyanobacteria

**DOI:** 10.1002/mbo3.1268

**Published:** 2022-03-16

**Authors:** Dorina Strieth, Selina Lenz, Roland Ulber

**Affiliations:** ^1^ Chair of Bioprocess Engineering University of Kaiserslautern Kaiserslautern Germany

**Keywords:** antimicrobial compounds, bioactivity assay, cyanobacteria, in silico screening, in vivo screening

## Abstract

Due to the emerging rise of multi‐drug resistant bacteria, the discovery of novel antibiotics is of high scientific interest. Through their high chemodiversity of bioactive secondary metabolites, cyanobacteria have proven to be promising microorganisms for the discovery of antibacterial compounds. These aspects make appropriate antibacterial screening approaches for cyanobacteria crucial. Up to date, screenings are mostly carried out using a phenotypic methodology, consisting of cyanobacterial cultivation, extraction, and inhibitory assays. However, the parameters of these methods highly vary within the literature. Therefore, the common choices of parameters and inhibitory assays are summarized in this review. Nevertheless, less frequently used method variants are highlighted, which lead to hits from antimicrobial compounds. In addition to the considerations of phenotypic methods, this study provides an overview of developments in the genome‐based screening area, be it in vivo using PCR technique or in silico using the recent genome‐mining method. Though, up to date, these techniques are not applied as much as phenotypic screening.

## Graphical abstract

Due to the emerging rise of multi‐drug resistant bacteria, the discovery of novel antibiotics is of interest. Through their high chemodiversity of bioactive secondary metabolites, cyanobacteria have proven to be promising microorganisms for the discovery of antimicrobial compounds. This review deals with the screening, including in vivo approaches like bioactivity assays as well as in silico approaches using contemporary genome‐mining tools in connection with cyanobacteria. The summarized tools are not only applicable for cyanobacteria and can be transferred to other microorganisms.

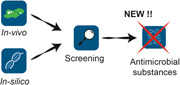

## 1 INTRODUCTION

The excessive use of antibiotics over the past decades has led to the rise of multi‐drug resistant (MDR) bacteria, making it one of the substantial problems faced by the modern health care system. Due to increased resistance, effective treatment becomes more and more complicated with the available, common antibiotics. Therefore, new treatments have to be brought onto the market, discovering new antibacterial substances, a key factor in the fight against the widespread of MDR bacteria (Laxminarayan et al., 2013; With, 2015).

Even though the pharmaceutical industry has made great advances in synthetic chemistry regarding the development of new, bioactive substances against a wide variety of pathogens, this technology still has its limitations: many natural products have highly complex structures that are too complicated and too expensive to produce on an industrial scale. In addition, natural sources offer a high diversity of substances, from which only a small part has been discovered so far. Therefore, the screening and isolation of bioactive compounds as new therapeutic substances remains an important aspect of research (Ahmad & Aqil, 2020; Lahlou, 2013).

In terms of bioactive compounds, cyanobacteria are a promising source of new, undiscovered substances. Cyanobacteria are photoautotrophic microorganisms that occur in many different environments, such as freshwater, seawater, and fields, leading to a high chemodiversity of secondary metabolites (Garcia‐Pichel et al., 2003; Swain et al., 2017). They produce a wide variety of bioactive compounds like proteins, lipids, polysaccharides, fatty acids, alkaloids, and polyketides, which are considered to have a variety of properties like antifungal, antiviral, antibacterial, algicidal, and anti‐inflammatory activity (Demay et al., 2019).

Due to the promising potential of cyanobacteria as producers of new bioactive compounds, a variety of reviews dealing with isolated substances have been published in the last few years (Levasseur & Pozzobon, 2020; Swain et al., 2017; Xue et al., 2018). Noticeably, these reviews focus on literature describing isolated and characterized compounds and do not provide information on the preceding screening leading to the discovery of antimicrobial substances from cyanobacteria. This review deals with the screening, including in vivo approaches like activity assays as well as in silico approaches using contemporary genome‐mining tools, extraction, and bioactivity assays used in connection with cyanobacteria. The summarized tools are not only applicable for cyanobacteria and can be transferred to other microorganisms.

## 2 SCREENING USING ANTIBACTERIAL ACTIVITY ASSAYS

**Figure 1:**
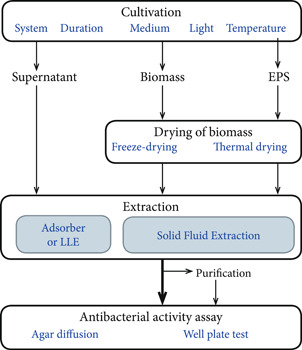
Schema of the commonly used procedure for the screening of antibacterial compounds from cyanobacteria, LLE, liquid‐liquid extraction; EPS, extracellular polymeric substances.

Conventional screening methods are based on cyanobacterial biomass. In most cases, the bioactive components are extracted from the dried biomass of the cultivation and then tested against bacteria using an in vivo activity assay to check for an inhibiting effect. The general schema of this procedure is provided in Figure 1. In general, it starts with the cultivation of cyanobacteria, which can vary in a variety of different parameters (light, temperature, medium, etc.). Inhibitory substances can then be extracted from the supernatant, biomass (including extracellular polymeric substances [EPS]). These extracts are then used for antibacterial activity assays. The following chapter deals with common cultivations, extraction conditions, and antibacterial activity assays, but also gives a brief outlook on less prevalent methods. An overview of cyanobacterial extracts with antibacterial properties and their respective method of cultivation, extraction, and activity assay are given in Table 1.

**Table 1:** Overview of the antimicrobial activity of cyanobacterial extracts, as well as extraction parameters (fraction of the cultivation, solvent, and special properties of the extraction), antimicrobial activity assay, and cultivation parameters (culture temperature/media/duration/and light intensity/light‐dark‐rhythm)

Source	Cyanobacterium strain	Antimicrobial activity against	Cultivation		Extraction				Antimicrobial Assay	
			Conditions and duration	Light intensity/light‐dark rhythm	Tested fraction	BM drying	Extraction solvent		Assay type	Parameter
Nainangu et al., 2020	*Oscillatoria* sp. *SSCM01*	STY, SA, EC, KP	25°C, BG‐11 N+; pH 7.4, 30 days	40–50 µmol Photons/(m²s), 14/10	Crude extract + fractions	Not stated	1:1 methanol: chloroform		Disc diffusion + resazurin assay	37°C, 24 h
	*Phormidium* sp. *SSCM02*	STY, SA								
Vasudevan et al., 2020	*Microcystis aeruginosa*	EC, SA, BS, VH, VC, PA	Direct sample		Crude extract	Not stated	Methanol		Disc diffusion	37°C, 24 h
Yalcin et al., 2020	*Phormidium autumnale*	EC, SE, SA, SAG, EF	BG‐11, 25 C, 15 days	50 µmol photons/(m²s)16/8	Crude extract	Not stated	Methanol/acetone		Disc diffusion + micro dilution	24 h
Konstantinou et al., 2020	*Synechococcus*sp. *0815*	SA	20°C, BG‐11 medium (+nitrogen)	20 µmol photons/(m²s)12/12	Crude extract	FD	90% methanol		Disc diffusion	37°C, 48 h
	*Leptothoe sithoniana 0915*	SA								
	*Leptothoe spongobia 1115*	SA								
	*Pseudanabaena*cf. *persicina 1415*	SA, PA, EC								
	*Leptothoe kymatousa 1615*	SA								
Hassan et al., 2020	*Spirulina platensis*	EC, KS, SE, SA	No temperature control, BG‐11, 20 days		Crude extract	40°C	97% ethanol	soxhlet extractor	Well diffusion	37°C, 24 h
N. Padmini et al., 2020	*Oxynema thaianum ALU PBC5*	EC, KP	30°C, ASN‐III medium pH 7,4	2500 Lux, 14/8	Crude extract	60°C	Chloroform/acetone/dichloromethane/ethyl acetate/petroleum ether		Disc diffusion	37°C, 24 h
Shishido et al., 2020	*Fischerella* sp. *CENA71*	SA	20°C, Z8, 21–28 days	10 µmol photons/(m²s) constant	Crude extract	FD	1. methanol; 2. dichloromethane/water		Disc diffusion	35°C–37°C, overnight
	*Fischerella* sp. *CENA72*	SA								
	*Fischerella* sp. *CENA161*	SA								
	*Fischerella* sp. *CENA298*	SA								
	*Aliinostoc* sp. *CENA513*	SA								
	*Aliinostoc* sp. *CENA514*	SA								
	*Aliinostoc* sp. *CENA535*	SA								
	*Aliinostoc* sp. *CENA548*	SA								
Gkelis et al., 2019	*Microcystis flos‐aquae TAU‐MAC 1510*	EC, SA	20°C–25°C, BG‐11	25 µmol Photons/(m²s)12/12	Crude extract	FD	90% methanol		Disc diffusion	37°C, 48 h
	*Synechococcus* cf. *nidulans TAU‐MAC 3010*	SA								
	*Jaaginema* sp. *TAU‐MAC 0211*	EC, SA								
	*Calothrix epiphytica TAU‐MAC 0399*	SA								
	*Limnothrix redekei TAU‐MAC 0310*	EC, SA								
Deyab et al., 2019	*Microcystic aerginosa*	KP, PA, SA	Direct sample	‐	Crude extract	Air‐dried	1. Methanol 2. Petroleum ether (3. Chloroform)		Disc diffusion	37°C, 24 h
Hemlata et al., 2018	*Michrochaete*	PA, EC, SA	30°C, BG‐11 pH 8	25 µmol Photons/(m²s)12/12	Crude extract	50°C	0.1 M potassium phosphate buffer (pH7.1)	repeated freezing and thawing	Micro dilution	37°C, overnight, 595 nm
Kumar et al., 2018	*Nostoc* sp.	EC	22°C, BG‐11		Crude extract	60°C	Methanol	sonic assisted	Well diffusion	37°C, 24–48 h
	*Limnothrix* sp.	EC								
	*Phormidium* sp.	EC								
Levert et al., 2018	*Lyngbya majuscula*	EC, ML	?		Pure substance	FD	Ethyl acetate		Microdilution	37°C, 24 h, 630 nm
Veerabadhran et al., 2018	*Leptolyngbya* sp. *AP3b*	EC	27°C	36–45 µmol Photons/(m²s)14/10	Crude extract	Not stated	1:1 chlorofom: methanol		Resazurin assay	37°C;18–24 h, 560/590 nm
	*Chroococcus* sp. *AP3U*	EC								
Cheel et al., 2018	*D. muscorum CCALA 125*	BS	28°C, 10 days		Partial purified extract	FD	Methanol + seasalt		Micro dilution	37°C, 16 h
Pham et al., 2017	various Nostoc sp. Isolates	SA, BS, SF, STY	BG‐11,7–8 weeks	12/12	Crude extract	FD	Ethyl acetate/methanol	sonic assisted	Disc diffusion	(4°C, 24 h) 37°C, 24 h
Belhaj et al., 2017	*Phormidiumversi‐color NCC 466*	EC, SA, ML, BA	25°C, modified BG‐11, 11 days	100 µmol Photons/(m²s)14/10	Polysaccharide extract	45°C	Water		Disc diffusion + MTT assay	(4°C, 2 h) 37°C, 24 h
Strieth et al., 2017	*Nostoc sphaeroides (*formerly *Trichocoleus sociatus)*	EC	24°C, BG‐11	100 µmol Photons/(m²s)	EPS extract	EPS; FD	0.14 M NaCl+ 0.2 M EDTA		Resazurin assay	
Hamouda Ali & Doumandji, 2017	*Spirulina platensis*	EC, KS, ST, PA	25°C, 5–6 days	7.5/10 µmol Photons/(m²s)16/8	Crude extract	60°C		Soxhlet extractor		(4°C, 2 h) 37°C,
										18–24 h
Barboza et al., 2017	*S. aquatilis M622C*	SA (methanol)	25°C, BG‐11/Conway	12/12	Crude extract	FD	Methanol or ethanol		Well diffusion	37°C, 18–24 h
	*Synechococcus* sp. *M94C*	PA (ethanol)								
	*Synechococcus* sp. *M290C*	PA (ethanol)								
	*R. gracilis M6C*	PA (ethanol + methanol)								
Elshouny et al., 2017	*Spirulina platensis*	EC, SAS, SHS, SA, PA	30°C, Zarrouk/Kuhl, until late exponential phase		Crude extract	60°C	Methanol, ethanol, ethyl acetate, and chloroform	sonic assisted	Microdilution + well diffusion	37°C, 24 h, 620 nm
	Different isolates, not specified	EA, YE, (BC, LM, ML, PA, SA)	BG‐ 11 agar, 2–3 weeks		Cyano‐bacteria	‐	‐		Agar inhibition	37°C, 24 h
Esquivel‐Hernández et al., 2017	*A. platensis*	SA, PA, EC (polar solvent)	Modified Jourdans, 8 days		Crude extract	Air‐dried	Ammonium acetate 10 mM and ethanol/limonene and ethyl acetate	microwave‐assisted	Disk diffusion	30°C, 24 h
A. Srivastava et al., 2017	*Phormidium CCC727*	EC EN, ST, SB, KP, EA	28°C, BG‐11	14–40 W/m^2^, 18/6	Crude extract	FD	Methanol; dissolved in methanol, acetone, DSMO, or diethyl ether		Micro dilution + disk diffusion	37°C, 24 h
	*Geitlerinema CCC728* sp.	EC, SA, EN, ST, SB, KP, EA								
	*Phormidium CC731*	EC, SA, SB								
	*Arthrospira CCC729*	EC, EN, ST, SB, KP, EA								
	*Leptolyngbya CC732*	EC, EN								
	*Phormidium CCC730*	EC, SA, EN, EA								
Montalvão et al., 2016	*Geitlerinema* sp.	EF	22°C, 23 days	100 µmol Photons/(m²s)constant	Crude extract	FD	80% ethanol			
Niveshika et al., 2019	*Nostoc* sp. *MGL001*	EC, PV, PA	25°C, BG‐11, 40–45 days	95 µmol Photons/(m²s)14/10	Pure substance	FD	Methanol		Disk diffusion	37°C, 24 h
Costa et al., 2015	*Cyanobium* sp.	PP	25°C, Z8 + 20 g/L NaCl,	30–40 µmol Photons/(m²s)14/10	Crude extract/fractions	FD	1:2 methanol: dichloromethane		Microdilution	25°C, 24 h, 750 nm
Lamprinou et al., 2015	*T. calypsus*	SA, SA (MRSA), SA (MSSA), EF, EF(VRE) and EF (VRE)	23°C, BG‐11/BG‐11 0, 150–200 days	7 µmol Photons/(m²s)	Lipid fractions	Not dried	Bligh Dyer method (1:2 chloroform/methanol, + chloroform + water)		Disk diffusion + micro dilution	37°C, 24 h
	*P. melanochroun*	SA, SA (MRSA), SA (MSSA), EF, EF(VRE) and EF (VRE)			Lipid fractions					

Abbreviations: BA, *B. amyloliquefaciens*; BC, *B. cereus*; BM, Biomass; BS, *B. subtilis*; EA, *E. aerogenes*; EC, *E. coli*; EF, *E. faecalis*; EN, Enterococcus; FD, freeze‐dried; KP, *K. pneumoniae*; KS, *Klebsiella* sp.; LM, *L*. *m* *onocytogenes*; PA, *P. aeruginosa*; PP, *Pseudomonas putida*; PV, *P. vulgaris*; SA, *S. aureus*; SAG, *S. agalactiae*; SAS, *Salmonella* sp.; SB, *S. boydii*; SE, *S. epidermidis*; SF, *S. flexneri*; SHS, *Shigella* sp.; ST, *S. typhimurium*; STY, *S. typhi*; VC, *V. cholerae*; VH, *V. harveyi*; YE, *Y. enterocolitica*.

### 2.1 Enhanced production of antimicrobial compounds by varying cultivation parameters

**Figure 2:**
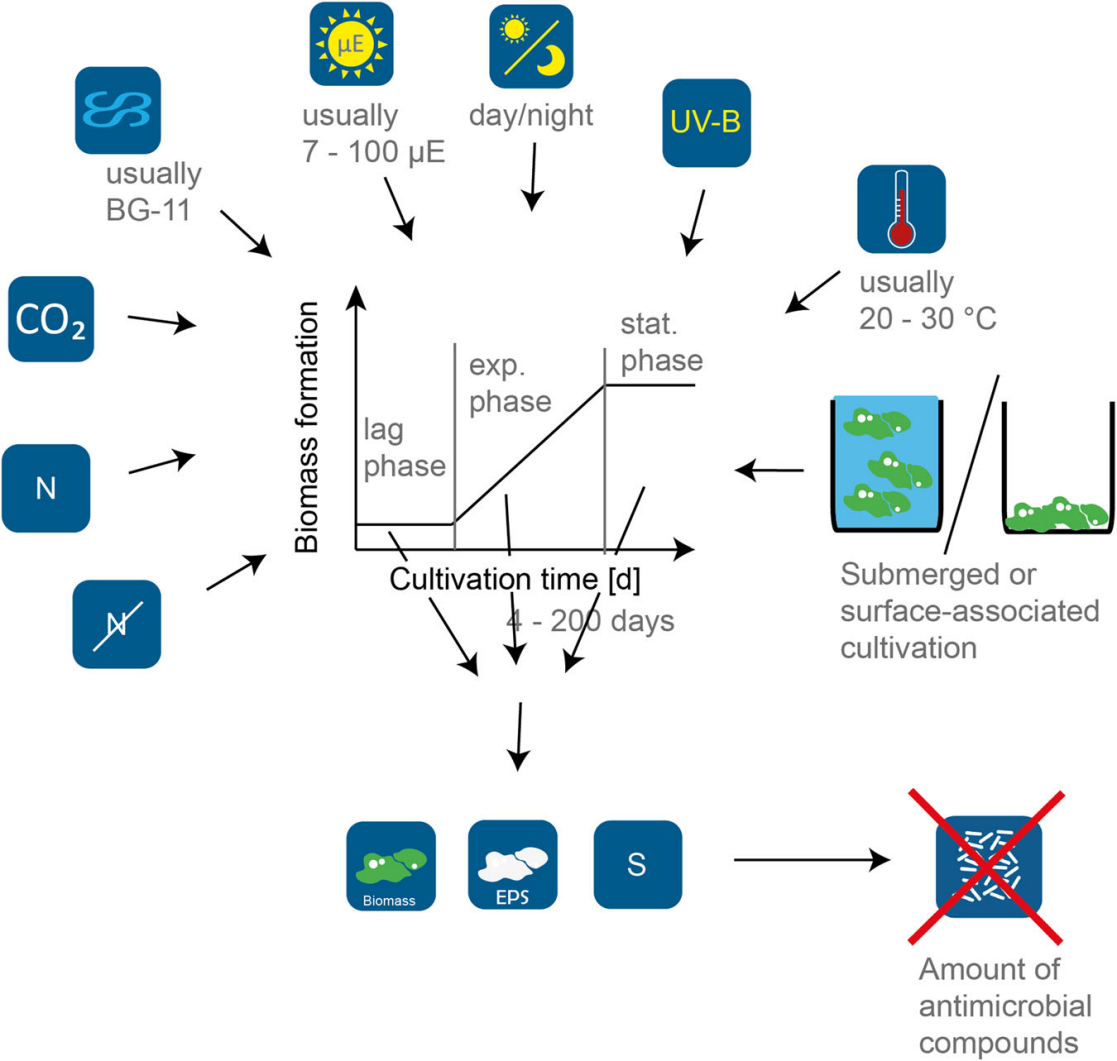
Schematic overview of cultivation parameters that can influence the production of antimicrobial compounds. N, nitrogen; S, supernatant; EPS, extracellular polymeric substances; exp., exponential; stat., stationary; µE, mmol photons/(m^2^s)

Environmental samples can be screened directly by using them for extraction and a subsequent antimicrobial activity assay (Deyab et al., 2019). However, if an interesting compound is detected larger amounts of biomass are often required for the extraction and further characterization of the unknown substance. Therefore, the natural consortium can be cultivated in special bioreactors imitating the natural habitat, or the cyanobacteria have to be isolated. However, for further investigations, high biomass productivity and high production of antimicrobial compounds are required. The cultivation parameters of this step can differ greatly (see Table 1). Temperature is normally chosen between 20°C and 30°C and the light intensity in the reviewed literature ranges from 7 up to 100 µmol Photons/(m²s) (Belhaj et al., 2017; Lakatos & Strieth, 2017; Lamprinou et al., 2015; Montalvão et al., 2016). In some instances, a constant light source, and in some instances a day/night cycle of different lengths were simulated (see Table 1). Cultivation is commonly conducted as photoautotrophic cultivation submerged in standard media such as BG‐11 with or without nitrogen (Rippka et al., 1979) or Z8 (Kotai, 1972). In general, the cultivation conditions likely reflect default methods for the cultivation of cyanobacteria and no specific strategy designed to optimize the production of antimicrobial compounds. Exceptions are, for example, the cultivation of the terrestrial cyanobacterium *Nostoc* sp. (formerly *Trichocoleus sociatus*) in an aerosol‐based photobioreactor, leading to a substantial increase of the antimicrobial activity in comparison to submerged cultivation (Strieth et al., 2017). The exposure of cyanobacterial cultures to UV‐B radiation leads to a decreased minimum inhibitory concentration (MIC) of the resulting crude extract (Fatima et al., 2017). One parameter of particular interest is the cultivation time until harvest for the antibacterial activity assay since the content of an antimicrobial compound can change over‐cultivation (Chetsumon et al., 1993). For cyanobacterial cultures, comparatively long cultivation times are common. The cultivation duration varied between 4 and 200 days. The duration of 150–200 days described by Lamprinou et al. (2015) was stated to be necessary for the production of sufficient biomass. However, a very low light intensity of 7 µmol Photons/(m^2^s) was used, which likely led to a low growth rate, since light conditions strongly influence biomass productivity (Lakatos & Strieth, 2017). Nevertheless, the tolerable exposure intensity differs greatly between different cyanobacteria and needs to be taken into account (Lamprinou et al., 2015). Besides the light intensity and other cultivation parameters, the phase of harvesting the biomass varies within the literature. In many papers biomass from the exponential phase was used (Elshouny et al., 2017; Konstantinou et al., 2020; N. Padmini et al., 2020), which is reached after different cultivation durations, depending on the growth speed of the corresponding cyanobacteria. Hamouda Ali and Doumandji explicitly stated that biomass was harvested before reaching the exponential phase, namely after 5–6 days (Hamouda Ali & Doumandji, 2017). Figure 2 gives an overview of the different cultivation parameters that can influence the production of antimicrobial compounds.

### 2.2 Extraction

**Figure 3:**
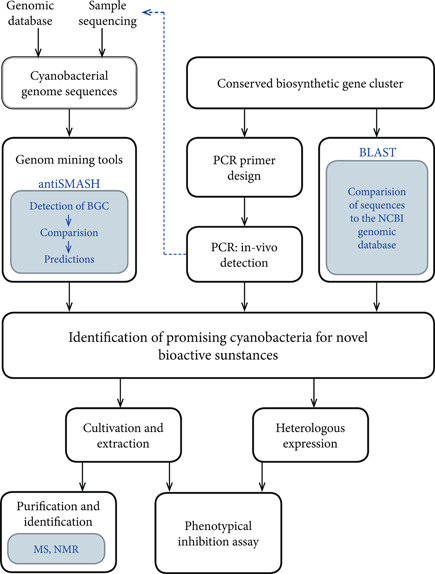
Different approaches for the usage of genome‐based screening methods for the identification of promising cyanobacteria for novel bioactive substances, using in vivo and in silico tools as well as Mass spectrometry (MS) and nuclear magnetic resonance (NMR) for purification

One of the difficulties in extracting an unknown substance is choosing the most suitable extraction solvent without knowing the properties of the compound, such as polarity, and so on. A good solvent for the extraction of antimicrobial activity preferably has a relatively low boiling point, to simplify removal, and does not interfere with the subsequent activity assay, since residues of the solvent may remain in the dried extract. Throughout the literature, a large spectrum of polar and nonpolar solvents, as well as their mixtures are used for the extraction of antimicrobial substances, like methanol, acetone, ethyl acetate, ethanol, petroleum ether, chloroform, isopropanol, and water (see Table 1). Since the substances to be extracted are unknown, different extraction solutions should be used at the beginning, and the antibacterial activity should be tested and compared (Figure 3).

(Barboza et al., 2017; Esquivel‐Hernández et al., 2017; N. Padmini et al., 2020). Esquivel‐Hernandez et al. for example, tested polar and nonpolar solvents for the extraction (Esquivel‐Hernández et al., 2017). The polar extract of *Arthrospira platensis* showed high antimicrobial activity against Gram‐positive bacteria (*Staphylococcus aureus*) and Gram‐negative bacteria (*Pseudomonas aeruginosa*, *Escherichia coli*), while the non‐polar extract only indicated a moderate activity against *P. aeruginosa* and *E. coli*. In the study of Pham et al, only the extract using methanol was antibacterial active and not the ethyl acetate extract (Pham et al., 2017). Fatima et al. compared water, isopropanol, and methanol for extraction and tested the activity of these extracts against *Staphylococcus leopoliensis* (Fatima et al., 2017). The MIC of the methanol extract was around 50% lower than that of the isopropanol or water extract. Interestingly, the methanol extract worked against all tested bacteria strains (*E. coli, S, aureus, K. pneumoniae, P. aeruginosa*, and *E. aerogenes*), while the aquatic extract only inhibited the growth of *E. coli, S. aureus*, and *E. aerogenes*. Thus, it can be assumed that more than one active substance is produced in this case (Fatima et al., 2017). Methanol is one of the most commonly used solvents and also shows to be one of the most efficient solvents regarding the antimicrobial activity of the resulting extract. In general, polar solvents seem to be more suitable for the extraction of bioactive compounds (Barboza et al., 2017; Esquivel‐Hernández et al., 2017). Using different polarities of solvents can help to increase the purity of the extract. This method was applied by Hamouda Ali and Doumandji who successively extracted dry biomass from the cyanobacterium *Spirulina platensis* with diethylether hexane, dichloromethane, and acetone. Each extract showed different effects in the inhibition of bacterial growth, whereby the diethyl ether hexane extract had the highest antibacterial activity (Hamouda Ali & Doumandji, 2017).

Commonly, the dried cyanobacterial biomass (BM) including the EPS is used for extraction. Variations in the preparation of the extraction start with the drying of the biomass. Since an unknown substance is to be extracted and no statement regarding its heat resistance can be made, lyophilization is a popular choice (Gkelis et al., 2019; Levert et al., 2018; Montalvão et al., 2016). However, drying processes up to 60°C are used as well (Elshouny et al., 2017; Hamouda Ali & Doumandji, 2017).

As an alternative to the extraction from biomass, bioactive substances can also be extracted from different shares of cyanobacterial cultivation: the EPS or the cultivation supernatant. Though, these approaches are relatively rare in screening. One example is Lamprinou et al. using undried biomass for extraction and another is Strieth et al. using EPS (Lamprinou et al., 2015; Strieth et al., 2017). The concept of using the supernatant for extraction is not well established in the screening of cyanobacteria, although it is already used more frequently in other areas (Moradi et al., 2019; Thomas Hoffmann et al., 2018). This extraction type is based on the assumption that an antimicrobial substance, which is produced as a defense mechanism, can also be secreted (Alkotaini et al., 2013; R. A. Mogea et al., 2015). In general, extraction using the supernatant can be done by liquid‐liquid extraction or solid‐phase extraction (SPE) using different resins. Cheel et al. used a XAD Amberlite resin to enrichen the crude extract from cyanobacterial biomass (Cheel et al., 2018).

In general literature, a large variety of different liquid‐liquid and solid‐liquid, extraction methods are described like ultrasonic‐assisted extraction, solvent microextraction (SME), supercritical fluid extraction (SFE), and pressurized liquid extraction (PLE) (Bendicho & Lavilla, 2000; Kim et al., 2014; Kokosa, 2014). Interestingly, the extraction methods used for the screening of antibacterial compounds from cyanobacteria are relatively basic. Most of the time, extraction is conducted as a solid‐liquid extraction, by simply immersing the dried biomass in extraction solvent, often supported by prior grinding using a mortar. Occasionally, a microwave or sonic‐assisted extraction is applied (Elshouny et al., 2017; Esquivel‐Hernández et al., 2017; Pham et al., 2017; Kumar et al., 2018) or a Soxhlet extractor is used (Hamouda Ali & Doumandji, 2017; Hassan et al., 2020). Soxhlet extraction allows the matrix to be in contact with fresh solvent over the whole process, while sonic‐assisted extractions promote cellular disruption and are reported to achieve remarkably high yields and extraction rates for bioactive compounds (Osorio‐Tobón, 2020). Extraction can also be encouraged by repeated freezing and thawing. This procedure can lead to the destruction of antimicrobial compounds, depending on their stability (Hemlata & Fatma, 2018).

### 2.3 Antimicrobial activity assay

A good activity assay is crucial for a successful in vivo screening for antimicrobial substances. Ideally, an assay is cheap, easy, has fast/high‐throughput, and has high sensitivity as well as reproducibility. Furthermore, it needs to be ensured that no compounds of the extract are interfering with the assay itself (Hadacek & Greger, 2000). The antimicrobial activity of an extract or substance can be determined using several different assays, with the most common being the agar diffusion and microdilution assay.

For the agar diffusion assay, a culture of a bacterial test strain (e.g., *E. coli*) is prepared and uniformly spread on an agar culture plate. The extract is then applied to the plate with a disk (disk diffusion test) or wells are punched into the agar and filled with extract (well diffusion test) (Bonev et al., 2008). After incubation of the agar plates, they can be examined for an inhibition zone around the discs or wells, where an antimicrobial compound diffusing into the agar would inhibit bacterial growth. The antibacterial activity of the extract can then be described using the size of the inhibition zone, with a larger inhibition zone corresponding to a higher antibacterial activity (Bonev et al., 2008). Official manuals for carrying out inhibition tests are described by the European Committee on Antimicrobial Susceptibility Testing (EUCAST) or Clinical and Laboratory Standards Institute (CLSI, formerly known as National Committee for Clinical Laboratory Science (NCCLS). Since screening does not need to comply with official directives, the actual execution of these assays will often vary, concerning the incubation temperature (30°C–37°C) (Hemlata & Fatma, 2018; Nainangu et al., 2020), incubation time (overnight up to 48 h (Gkelis et al., 2019; Shishido et al., 2020), or a preceding incubation at low temperatures to allow the extract to diffuse into the agar without promoting bacterial growth (Belhaj et al., 2017; Hamouda Ali & Doumandji, 2017; Pham et al., 2017). One challenge, which hinders the comparison of inhibition zones between different papers, is the high variance in the amount of used extract, as well as the varying extract concentration and concentration of the antimicrobial compound within the crude extract.

As an alternative to the agar diffusion assay, inhibition can also be examined using well plate‐based assays, in which the inhibition is usually anti‐proportional to an increase in the optical density of a bacterial test strain. Alternatively, a well plate test can be conducted as a resazurin assay, in which resazurin is enzymatically reduced to resorufin by hydrogenases using NADH/NADPH as co‐substrate and causing a shift of fluorescence wavelength (Präbst et al., 2017). The resazurin assay is proclaimed to have an advantageous sensitivity compared to optical density‐based tests (Palomino et al., 2002). If the bioactive substance is applied in a variety of concentrations, the assay is called microdilution and the inhibition can be described by the MIC, describing the lowest concentration inhibiting visible bacterial growth. Sometimes the inhibition is additionally stated using the minimum bactericidal concentration (MBC), which describes the lowest concentration needed to kill a bacterium. To obtain the MBC, the respective bacteria are sub‐cultured after performing an inhibition assay to obtain the capacity of reproduction (Owuama, 2017). Alternatively, the antibacterial activity can be described using an 'inhibition percentage', which is based on positive (commercial antibiotics) and negative controls (buffer or media). In comparison to an agar diffusion assay, a microdilution assay has the advantage of commonly describing the MIC, in which the concentration is directly implied, reducing variations between different working groups. In addition, a microdilution assay can be carried out in a well plate, allowing a significantly higher throughput than an agar method. The conditions for the assay vary in a similar way to the agar diffusion assay with different incubation times (overnight up to 24 h) and incubation temperature (25°C–37°C). Furthermore, optical density can be measured at different wavelengths (Costa et al., 2015; Levert et al., 2018).

Even though there are a variety of assays available, most of the time agar diffusion or microdilution assay measuring the optical density is used, since these methods are already well established in most laboratories. Even though the inhibition zone assay has drawbacks like its expenditure of time, low accuracy, and detection limit, it is a simple, cheap, and robust method that can be carried out in practically every laboratory since little specific equipment is required (Osato, 2000).

No matter which test is chosen different parameters can influence the results:

- The time point at which the antimicrobial substance is added.
- Time and temperature of diffusion of the antimicrobial substance.
- Inoculum concentration of test strains.
- Test strain itself.
- Incubation time before measurement.
- Co‐extracted compounds can disturb especially fluorescence or colorimetric assays.
- Amount of antimicrobial compounds.
- Purity of antimicrobial compounds.
- Extraction solution.

Every bioactivity assay has advantages, disadvantages, and needs to be chosen based on the laboratory equipment. The biggest issue when comparing the achieved results with the literature is that most of the researchers use the method and parameters that are established at their institute. There is no general comparison of the available bioactivity methods since the detection of an inhibitory effect differs extremely. A key question during screening is at which point an antibacterial effect is classified as significant. Most papers only provide an overview of the resulted inhibition zones and highlight their most effective extracts. This approach, however, only compares inhibition properties to other results from the own screening and leaves the reader guessing, which of the obtained inhibition zones can be considered significant. As already stated, the comparison of inhibition zones is difficult due to varying concentrations, but some papers at least state boundaries of their evaluation of the inhibitory effect of the crude extracts. One example for such an evaluation stated by Belhaj et al. is Ø ≤ 7 mm: no antimicrobial activity; 7 mm ≤ Ø ≤ 9.9 mm: low antimicrobial activity; 10 mm ≤ Ø ≤ 11.9 mm: modest antimicrobial activity; 12 mm ≤ Ø ≤ 15 mm: high antimicrobial activity; 15 mm < Ø: strong antimicrobial activity. For comparison, within the paper an inhibition zone of 7 mm corresponding to a MIC of 2.5 mg/ml; one of 12 mm to a MIC of 0.16 mg/ml, and one of 15 mm to a MIC of 0.08 mg/ml (Belhaj et al., 2017). Although this approximation needs to be viewed with caution as the inhibition zone assay is also dependent on the diffusion rates of the compound, which are highly determined by the polarity of the substance (Ncube et al., 2008). If the limits of Belhaj et al. would be assumed for other screenings, for example, the extract of *Nostoc* sp. or *Phormidium* sp. described by Kumar et al. would be considered to have no inhibitory effect, since the inhibition zone was only around 6 mm (Kumar et al., 2018).

#### 2.3.1 Test strains

A wide range of gram‐negative and gram‐positive bacterial strains are used for the assays. The extent of different testing organisms differs within the literature. Sometimes, only one strain was used for testing, sometimes a range of up to eight strains. A list of the used bacteria from the viewed literature is listed in Table 1.

The most common strains include *S. aureus*, *E. coli*, and *P. aeruginosa*. The general selection of the strains also reflects the clinical importance of the bacterial strains. *Klebsiella*, *Staphylococcus*, and *Pseudomonas* are genera of pathogenic bacteria, which can lead to a variety of infectious diseases, with *S. aureus* being the most pathogenic of the genus *Staphylococcus* (Azam & Khan, 2019; Pérez‐Montarelo et al., 2017; Podschun & Ullmann, 1998). Bacteria of the genera *Shigella* and *Salmonella*, as well as *E. coli*, are known food pathogens that can cause serious food poisoning (Dolman, 1943; FDA, 2020). Additionally, *S. aureus* and many bacteria from the genus *Pseudomonas* have known strains that are resistant to commonly used antibiotics (Köck et al., 2010; Pang et al., 2019). In response to that, some activity assays are testing the antibacterial activity of the extract against antibiotic‐resistant strains like Vancomycin‐resistant *E. faecium* (VRE) and Methicillin‐resistant *S. aureus* (MRSA). Even against these, some extracts from cyanobacteria were able to achieve an inhibiting effect (Lamprinou et al., 2015).

Within the literature, there is no clear trend if extracts from cyanobacteria are more effective against gram‐positive or gram‐negative bacteria. This indicates a great diversity of the different substances and associated mechanisms of action. Sometimes extracts are only effective against a certain type of bacterium, but often they can yield an activity against a whole range of bacteria (Hamouda Ali & Doumandji, 2017; Vasudevan et al., 2020; Yalcin et al., 2020). Since cyanobacteria can synthesize more than one antibacterial molecule, an extract of the same strain may also differ in its activity against different bacteria depending on the extraction solvent. For example, the aqueous extract obtained from *Synechococcus* spp. inhibited the growth of *S. aureus*, *K. pneumoniae*, and *E. aerogenes*, while the extract using isopropanol and methanol inhibited the species listed above as well as *E. coli* and *P. aeruginosa* (Fatima et al., 2017). In general, the type of bacteria used for antimicrobial assays may also depend on the location of the laboratory since the handling of pathogenic strains is controlled by national laws, dealing with the prevention and control of infectious diseases.

## 3 GENOMIC APPROACHES FOR THE SCREENING

Due to the phenotypic nature of traditional screening methods, they rely on the synthesis of a sufficient amount of antibacterial components during cyanobacterial cultivation to be able to detect it in a subsequent inhibition assay. Since cyanobacteria grow rather slowly, this can lead to a long cultivation time before an activity assay is possible (Lamprinou et al., 2015; Niveshika et al., 2019; Pham et al., 2017). In addition, cultivation conditions have a high impact on the production of secondary metabolites. As a consequence, promising candidates for new antibiotics might be neglected due to unsuited cultivation conditions, leading to a decreased production of secondary metabolites. Therefore, the interest in genome‐based screening as an addition to the phenotypic screening of cyanobacteria has increased in recent years (Micallef, D'Agostino, Al‐Sinawi, et al., 2015; Micallef, D'Agostino, Sharma, et al., 2015; Singh et al., 2010). This interest was mainly promoted by the fact that the availability and accessibility of genome data have highly improved. In combination with the creation of new bioinformatics tools, this has generated many new options for screening (Corre & Challis, 2007; Levasseur & Pozzobon, 2020; Shiha et al., 2013). In general, genomic methods can be divided into molecular biological methods, using for example polymerase chain reaction (PCR) for the detection of DNA sequences in vivo, or genome mining approaches in which genomic data are analyzed in silico.

### 3.1 Properties of antibacterial gene clusters

For the discovery of new bioactive substances based on genomic properties, significantly more information than for the execution of an antibacterial test is needed. It is, therefore, crucial to examine data about similar substances and their related biosynthesis from literature. There are several reviews about cyanobacteria dealing with the properties of already isolated and characterized substances and their corresponding bioactive activities (Agrawal et al., 2017; Tan & Phyo, 2020). Cyanobacteria are described to synthesize a range of antibacterial substances from different substance classes: alkaloids, depsipeptides, lipopeptides, macrolides/lactones, peptides, terpenes, polysaccharides, lipids, polyketides, and others (Swain et al., 2017). A majority of these bioactive substances are described to be peptide‐derived. Peptide‐derived compounds can be synthesized through nonribosomal peptide synthetases (NRPS), polyketide synthases (PKS), or as ribosomal synthesized and post‐translationally modified peptides (RiPPs). Mixing routes of NRPS/PKS are also described (Agrawal et al., 2017; Swain et al., 2017). NRPS and PKS are multifunctional enzymes that are organized in modules with an approximate size of 200–2000 kDa (Ehrenreich et al., 2005). An example of antibiotic active substances synthesized in this way is Brunsvicamide B and C, from the cyanobacterium *Tychonema* sp. The cyclic hexapeptides can selectively inhibit the *Mycobacterium tuberculosis* protein tyrosine phosphatase B (MptpB), therefore making it a promising treatment against *M. tuberculosis* (Müller et al., 2006).

### 3.2 Screening using genome mining and PCR

In general, most of the secondary metabolites are synthesized via bioactive gene clusters (BGC) (Naughton et al., 2017). These gene clusters often contain highly conserved sequences within a substance family, such as the adenylation modules of the NRPS or LanC, which is involved in the modification of lantibiotics (Mayer et al., 2001; Shiha et al., 2013). A conserved sequence refers to a nucleotide sequence with a very high homology across different species (Sarkar et al., 2011). The in silico screening for BGC is commonly called genome mining, which is described as the process of deriving information over an organism or its synthesized products through the analysis of genomic data and can be used for “predicting and isolating natural products based on genetic information without a structure at hand” (Ziemert et al., 2016). Genome mining can be done using a variety of different approaches. If the genome sequence of cyanobacteria is known (accession e.g., via NCBI (https://www.ncbi.nlm.nih.gov/), with up to date 500 complete genome sequences) it can be analyzed using web‐based genome mining tools. One well‐known tool is the “Antibiotics and Secondary Metabolite Analysis SHell,” commonly known as antiSMASH (Weber et al., 2015). This tool allows to identify gene clusters within a nucleotide sequence, as well as comparing them to known biosynthetic gene clusters (BGCs) to determine the gene cluster type as well as predict a possible product. Alternatives tools include BActeriocin GEnome Mining tooL (BAGEL), Evo Mining, and RODEO, contributing a high variety depending on the planned investigation (Weber, 2020; Secondarymetabolites.org) provides a good overview of the different tools that can be used for different approaches to investigate secondary metabolites or their corresponding gene clusters (Weber, 2020). On the other hand, conserved biosynthesis gene sequences (e.g., from NRPS or LanC) can also be used to search for genomes with highly similar sequences via BLAST (Basic Local Alignment Search Tool) from NCBI (Sandiford, 2017). In this way, cyanobacteria from a genome database can be screened regarding their possession of genomic sequences for the production of specific secondary metabolites. An example of the application of genome mining methodology was conducted by Micallef et al. using antiSMASH for the detection of biosynthetic gene clusters in subsection V cyanobacteria (Micallef, D'Agostino, Al‐Sinawi, et al., 2015). A putative gene cluster of the cyclic dipeptide hapalosin could be detected in three different cyanobacteria strains (Micallef, D'Agostino, Al‐Sinawi, et al., 2015). Vestola et al. described the biosynthetic pathway of an antifungal glycolipopeptide in *Anabaena* sp. SYKE748A, and was able to detect an antifungal variant of said glycolipopeptide in 4 other cyanobacterial genera (Vestola et al., 2014). Pancrace et al. discovered the antifungal Hassallidin E of *Planktothrix serta* PCC 8927 using antiSMASH 3.0 (Pancrace et al., 2017). Unfortunately, even with the rapidly increasing number of accessible genomes, only a small part of the naturally occurring cyanobacteria has been sequenced (NCBI Taxonomy, 2020).

If the genome of cyanobacteria is not sequenced, analysis can also be conducted in vivo by PCR. PCR is used to detect gene sequences within the genome through specific short nucleotide sequences called primers, which bind to complementary sequences and allow amplification of the DNA segment between forward and reverse primer by a DNA polymerase. There is also the possibility of designing a degenerated primer, which is a mixture of primers with highly similar sequences but substitution of different bases at some points of its sequence, making it possible to detect conserved regions of biosynthesis clusters in vivo (Sarkar et al., 2011). For example, this method was carried out by Ehrenreich et al., who examined isolated cyanobacteria for the presence of NRPS/PKS gene clusters to compare them with the cytotoxicity of the strains (Ehrenreich et al., 2005). Additionally, PCR products can be sequenced and used for further in silico analysis. This approach was used by Micallef et al. to close potential gaps in the nucleotide sequences (Micallef, D'Agostino, Sharma, et al., 2015)

Even if these approaches offer many new possibilities, they should be seen as an addition to phenotypic tests and are not capable of replacing them completely. For example, PCR can be used to detect NRPS gene clusters, which can lead to the synthesis of an antibacterial peptide. However since around 70% of the cyanobacteria contain a corresponding gene cluster, this information alone does not guarantee an antibacterial activity (Neilan et al., 1999). Hence, further investigations of antibacterial substances after the first molecular biological or genome mining approaches are crucial. The approaches are commonly coupled with a subsequent activity assay or isolation and analysis of the compound using mass spectrometry (MS) and nuclear magnetic resonance (NMR) to determine its structure (Mohimani et al., 2014; Sigrist et al., 2020). However, in silico methods have the advantage that the substance leading to a subsequent phenotypic hit is known, which greatly facilitates the purification. Partly, promising gene sequences are cloned into host bacteria like *E. coli* for a heterologous expression of the target molecule. The resulting extracts can then be screened using inhibition assays (Shi et al., 2019; Shih et al., 2013; Singh et al., 2010). However, it must be noted that nonphenotypic methodologies for the identification of bioactive substances in cyanobacteria are up to date a very small share compared to phenotypic screenings. Even today, genome mining in cyanobacteria is more of a promising outlook than a technique that is solidly established in most scientific institutes. Though, this could change as genomic data of cyanobacteria gets more available. One project to extend the coverage of cyanobacterial genome sequences is a cooperation of the University of Kaiserslautern and the University of Dresden that was awarded a whole‐genome sequencing grant from the Joint Genome Institute (JGI), USA. As part of this project, the genomes of 40 different cyanobacteria are going to be sequenced (TU Dresden, 2021).

## 4 SUMMARY

Natural substances from cyanobacteria are a relevant source for novel antibacterial substances. Phenotypic assays are mostly conducted using a roughly similar procedure of cultivation, extraction, and a subsequent inhibition assay. Regardless of this, it is not possible to specify uniform screening conditions caused by many small variances between the individual parameters. In extractions, freeze‐drying and polar solvents are predominant. In the case of the activity assay, standard methods such as microdilution and agar diffusion assays are used most of the time, even if new methods based on resazurin have been introduced. One major difficulty remains in the comparison between the results of different papers to conclude which cyanobacterial strains are particularly active and which ones are only more active compared to the other tested strains. Throughout the literature there are many examples of cyanobacteria showing promising antibacterial activity, which can be investigated further for the discovery of antibacterial substances. Furthermore, genome‐based methods for the discovery of new bioactive substances including in vivo and in silico approaches have been introduced for cyanobacteria. Although these are very promising technologies for the addition to phenotypic screenings, at the moment these do not have the same status as purely phenotypic methods.

## CONFLICTS OF INTEREST

The authors declare no conflicts of interest.

## ETHICS STATEMENT

None required.

## AUTHOR CONTRIBUTIONS

**Dorina Strieth**: Funding acquisition‐Equal, Supervision‐Equal, Writing—review and editing‐Equal. **Selina Lenz**: Writing—original draft‐Equal. **Roland Ulber**: Funding acquisition‐Equal, Project administration‐Equal.

## Data Availability

Not applicable.
